# Evaluation of endometrioma size effect on ovarian reserve, embryo quality and pregnancy outcome after in vitro fertilization cycle; a cross-sectional study

**DOI:** 10.1186/s12905-023-02482-1

**Published:** 2023-06-21

**Authors:** Afsson Zareii, Elham Askary, Ameneh Ghahramani, Kefayat Chamanara, Alimohammad Keshtvarz Hesam Abadi, Azadeh Afzalzadeh

**Affiliations:** 1 Department of Obstetrics and Gynecology, School of Medicine, Infertility Research Center, Shiraz University Of Medical Sciences, Shiraz, Iran; 2 Department of Obstetrics and Gynecology, Shiraz University Of Medical Sciences, Shiraz, Iran; 3 Clinical Research Development Center of Nemazee Hospital, Department of Statistics, Shiraz University of Medical Sciences, Shiraz, Iran; 4 Obstetrics and Gynecology Office, Shahid Faghihi Hospital, Zand Avenue, Shiraz, 7134844119 Iran

**Keywords:** Endometriosis, AMH, IVF, Pregnancy complications

## Abstract

**Introduction:**

Investigation of endometrioma size and its laterality on the quality of the embryo in patients with endometrioma compared to healthy subjects.

**Materials and methods:**

In this retrospective and cross-sectional study, 70 patients with unilateral and bilateral endometrioma were recruited and compared with 70 age-matched infertile patients as the control group in terms of AMH before ovum pick-up, embryo quality as well as pregnancy outcome. Additionally, in the case group, we divided both unilateral (n = 32) and bilateral endometrioma patients (n = 38) into three groups based on the size of endometrioma. (1–3 cm, 3–6 cm, 6–10 cm)

**Results:**

There was no difference in terms of age, BMI, parity, and age of menarche between the case and control groups. Moreover, no significant difference was observed in the baseline level of AMH between the case 2.96 ± 2.72 ng/dl (0.21–11.3) and control 2.73 ± 2.39 (0.21–12.8) groups. (P = 0.59) There was also no significant difference concerning AMH level between unilateral 3.58 ± 3.20 ng/dl (0.21–12.8) and bilateral endometrioma 2.45 ± 2.14 (0.21 − 0.20) groups. In terms of the quality and number of embryos, there was no significant difference between the case and control groups. (P = 0.30) Although the AMH level decreased with the increase in endometrioma size, this difference was not statistically significant. (P = 0.07) There was no significant difference in terms of the embryo quality between the groups based on the size of endometrioma. (P = 0.77) In addition, no significant difference was observed between the case and control groups in the terms of birth weight and pregnancy complications, such as premature delivery, cesarean section rate, neonatal respiratory distress, jaundice, as well as hospitalization rate. Head circumference of the newborns was higher in the endometrioma group while their Apgar score was lower in the case compared to the control group.

**Conclusion:**

The presence of endometrioma by itself does not affect the main result of IVF procedures, including the number and quality of embryos and pregnancy outcome. Thus, IVF and embryo preservation and even pregnancy before surgery seem to be reasonable for endometriotic patients.

## Introduction

Endometriosis is seen in 8.1–12.8% of women in the reproductive age [1–3]. Among this population, 30–50% suffer from infertility and about 25–40% have superficial or deep ovarian endometrioma [4–7]. Endometrioma is seen in ultrasound as a round cyst with a thick wall and ground glass appearance. Ultrasound sensitivity in the diagnosis of endometrioma (OMA) is 83.3% [8]. The presence of endometrioma is associated with more advanced stages of endometriosis disease, which is a sign of disruption of normal pelvic anatomy in affected women [1, 2, 9–11]. Endometrioma is accompanied with mechanical pulling effect based on its size as well as its content (inflammatory markers and proteolytic enzymes). Furthermore, cellular degrading agents lead to fibrosis and smooth muscle metaplasia and decrease cortex specific stromal cell. Accordingly, oxidative stress in the normal tissues around endometrioma is much higher compared to that around the other benign cysts [12–14].

The presence of endometrioma at the time of ovum pick up reduces antral follicular count, as a result of which ovum retrieval is disturbed [15]. Numerous infertility specialists refuse to enter endometrioma due to the possibility of an abscess formation or missing an occult early stage of cancer. However, miss management has not been reported to date [16].

Despite the high prevalence of endometriosis in infertile women, the best treatment method for reducing the pain and recurrence and improving fertility outcomes still remain controversial.

Although cystectomy is considered as the method of choice for definitive diagnosis of endometrioma, recent research has shown that cystectomy before performing IVF does not improve the clinical pregnancy rate and causes a drop in ovarian reserve due to further damage to healthy ovarian tissues; in addition, removal of endometrioma of below 3 cm causes more damage to the ovarian tissue compared to those of a larger size [17]. Nonetheless, the results of meta-analysis of 33 studies indicated that live birth rate and cumulative pregnancy rate in cases with endometrioma are not different in comparison to healthy people [18].

There is scarce research on the comparison of the quality of embryos in patients with endometrioma and healthy people and also the effect of endometrioma size on embryo quality and pregnancy outcome.

The present study therefore aimed to investigate the size of endometrioma and its laterality on the quality of embryo in patients with endometrioma compared to healthy subjects of the same age.

## Material and method

The present study is a retrospective, cross-sectional study under the code of ethics IR.SUMS.MED.REC.1400.159. It includes patients referred to Hazrat Zainab Hospital due to infertility from April 2015 to April 2019.

In order to homogenize the studied population, some patients left out from the beginning and they were not considered among the study samples, including: a history of autoimmune, infectious or inflammatory diseases over the last three months prior to the ovum pickup, any type of malignancies, previous endometriosis surgeries, previous ovarian or pelvic surgeries, a history of spontaneous pregnancy without IVF procedure, polycystic ovary syndrome, severe male factor infertility, FSH > 10, AMH < 0.5, a history of abdomino-pelvic radiotherapy or chemotherapy for any reason, the presence of leiomyoma and adenomyosis simultaneously.

140 patients 18 to 37 age who had a history of infertility for up to 1 year were included in this study. Exclusion criteria was included unwillingness to continue participating in the study.

Herein, 70 patients with unilateral and bilateral endometrioma were recruited, whose ovarian involvement was diagnosed by a skilled gynecologist through ultrasound as the case group. There were on the other hand 70 age-matched infertile patients in the control group who did not have endometrioma or endometriosis and had undergone IVF for an unknown reason or tubal factor.

### Informed consent

was obtained from all the participants in the study. The demographic data, clinical records and ultrasound characteristics of all the patients with endometriosis were collected through their clinical records.

### Primary outcome

The number and quality of embryos in the case and control groups; the effect of unilateral or bilateral endometrioma and its size on the number and quality of embryos in the case group.

### Secondary outcome

Comparison of the primary AMH level in the case and control groups, along with the effect of unilateral or bilateral endometrioma and its size on the AMH level in the case group.

The embryo quality was divided into three groups:

Grade A: 6–8 cells embryos without fragmentation and blastomeres of the same size;

Grade B: 6–8 cells embryos, 30–50% fragmentation or unequal blastomeres;

Grade C: 6–8 cells embryos with > 50% fragmentation or unequal blastomeres [19].

### Statistical analysis

The data were analyzed with SPSS software version 22 and the rank variables were compared with k2 test or Fisher’s exact test while quantitative variables were compared with T- test or man Whitney test. Klomogorov Smirinoff test was used for checking the data distribution. Moreover, the quantitative results were reported as mean and standard deviation and rank variables as frequency and percentage. A P-value of below 0.05 was considered as the level of significance.

## Results

As shown in Table 1, there was no difference in terms of age, BMI, parity, and age of menarche between the case and control groups.

**Table 1:** Demographic data of both groups

Variable	Case (N = 70)		Control (N = 70)		P-value
	Mean (SD)	Min -Max	Mean	Min -Max	
Mean Age (SD)	(4.40)30.68	39 − 21	(4.06)31.04	39 − 22	*0.760
Mean Height (SD)	(6.79)159.71	170 − 147	(6.77)158.87	170 − 147	*0.440
Mean Weight (SD)	(8.99)65.82	77 − 40	(8.15)64.47	77 − 45	*0.155
Body Mass Index (kg/m2)	(3.95)25.95	35.17–16.02	(3.73)25.69	35.66–17.22	**0.569
Mean Age of Menarche (SD) year	(2.37)12.05	16 − 9	(1.81)12.41	16 − 9	*0.255
Mean Duration of Disease (SD) Year	(3.67)3.95	20.00–1.00			

*: Mann Whitney test, **: Independent Sample T-test

Five patients in the case group and six in the control group were hypothyroid. In the case group, two subjects had bicornuate uterus.

In the case group, 32 patients (45.7%) out of 70 had unilateral endometrioma and 38 (54.3%) had bilateral endometrioma. We divided both unilateral and bilateral endometrioma patients into three groups based on the cyst size, whose results are summarized in Table 2.

**Table 2:** Cyst size in the case group

Variable	Unilateral Endometrioma (N = 32)	Bilateral Endometrioma (N = 38)	Total (N = 70)
Mean size ± SD Cm	4.34 (2.04)	4.97 (2.25)	4.68 (2.17)
Size Classification, Number & %			
1–3 cm	6 (18.8)	5 (13.2)	11 (15.7)
3–6 cm	16 (50.0)	18 (47.4)	34 (48.6)
6–10 cm	10 (31.3)	15 (39.5)	25 (35.7)

There was no significant difference concerning Baseline AMH level between the case 2.96 ± 2.72 ng/dl (0.21–11.3) and control 2.73 ± 2.39 (0.21–12.8) groups. (P = 0.59)

In addition, no significant difference was seen in terms of AMH level between the unilateral 3.58 ± 3.20 ng/dl (0.21–12.8) and bilateral endometrioma 2.45 ± 2.14 (0.21 − 0.20) groups. All the above-mentioned data are summarized in Table 3.

**Table 3:** Comparison of the pre-pickup level of AMH level among the unilateral, bilateral OMA, and control groups

Variables	Unilateral Endometrioma (N = 32)		Bilateral Endometrioma (N = 38)		Control (N = 70)		P-value
	Mean (SD)	Min-Max	Mean (SD)	Min-Max	Mean (SD)	Min-Max	
Mean AMH Level (SD)	3.58(3.20)	0.21–12.80	2.43(2.14)	0.21–10.20	2.73(2.39)	0.21–11.30	*0.330

*: Kruskal Wallis Test

There was no significant difference in the quality and number of embryos between case and control groups, and even between unilateral and bilateral endometrioma cases with the controls in pairwise comparison. (P = 0.30) Table 4 represents the results of the comparison between the embryo quality and the number of embryos retrieved from all the patients.

**Table 4:** Comparison of the embryo quality and number of embryos retrieved from all the patients

Variables	Unilateral Endometrioma (N = 32) (N & %)	Bilateral Endometrioma (N = 38) (N & %)	Control (N = 70) (N & %)	P-value
Embryo Quality				0.616*
A B C	13(46.4) 13(46.4) 2(7.1)	15(40.5) 20(54.1) 2(5.4)	34(50.7) 32(47.8) 1(1.5)	
Number of Embryo				0.555*
0 1 2 3 4 5 6 7 10	4(12.5) 3(9.4) 5(15.6) 4(12.5) 9(28.1) 1(3.1) 2(6.3) 2(6.3) 2(6.3)	1(2.6) 1(2.6) 11(28.9) 6(15.8) 10(26.3) 1(2.6) 5(13.2) 2(5.3) 1(2.6)	3(4.3) 5(7.1) 14(20.0) 14(20.0) 18(25.8) 12(17.1) 3(4.3) 1(1.4) 0(0)	

*: Fisher Exact Test

There was no significance difference in terms of AMH level and embryo quality between different sizes of endometrioma. Based on Table 5, although the AMH level decreases with the rise in the endometrioma size, this difference was not statistically significant. (P = 0.07) There was no significant difference concerning the embryo quality between the groups based on the size of endometrioma. (P = 0.77)

**Table 5:** Comparison of the AMH level and embryo quality according to the endometrioma size

Variables	1–3 cm N = 5 (N & %)	3–6 cm N = 18 (N & %)	6–10 cm N = 15 (N & %)	P-value
Mean AMH Level (SD) Ng/dl	3.22(4.00)	2.65(2.11)	1.91(1.26)	0.077*
Embryo Quality				
A (N = 15) B (N = 20) C (N = 2)	1(6.7%) 4(20.0%) 0(0.0%)	8(53.3%) 8(40.0%) 1(50.0%)	6(40.0%) 8(40.0%) 1(50.0%)	0.773**

*: Kruskal Wallis Test; **: Fisher Exact Test

Table 6 summarizes pregnancy outcome based on the case (unilateral and bilateral endometrioma) and control groups. There was no significant difference between the case and control groups in the terms of birth weight and pregnancy complications, such as premature delivery, cesarean section rate, neonatal respiratory distress, jaundice, as well as hospitalization rate. Head circumference of the newborns was higher in the endometrioma group whereas the Apgar score was lower in the case compared to that in the control group. As demonstrated, no significant difference was observed between the groups.

**Table 6:** Comparison of pregnancy outcomes in the endometrioma patients and control group

Variables	Unilateral N = 32 (N & %)	Bilateral N = 38 (N & %)	Control N = 70 (N & %)	P-value
Pre-term Labor	3(9.4)	4(10.5)	5(7.1)	0.925*
Respiratory Distress	2(6.3)	4(10.5)	9(12.9)	0.636*
Neonatal Admission	6(18.8)	9(23.7)	10(14.3)	0.480**
Jaundice	10(31.3)	13(34.2)	17(24.3)	0.513**
Mean Birth Weight, gram (SD)	2882.88 (276.05)	2866.62 (267.05)	2939.70 (338.97)	0.644†
Mean Head Circumference, cm (SD)	34.81(1.22)	34.69(1.11)	34.16(1.61)	0.046††
Apgar Score (5 min)	8.84(0.954)	8.65(0.937)	8.96(1.13)	0.081††

*: Fisher Exact Test, **: Chi-Square Test, †:One Way ANOVA, ††:Kruskal Wallis Test

## Discussion

In our age-matched retrospective study, there was no significance difference in the AMH level neither between the case and control groups nor the unilateral and bilateral endometrioma subgroups. The quality and number of embryos were the same in the case and control groups. Despite the decrease in AMH level with a rise in the size of endometrioma, this decrease was not statistically significant and the size of endometrioma did not therefore have a significant effect on the quality and number of embryos.

A few studies have investigated the effect of laterality and size of endometrioma on the quality and number of embryos. Most previous papers have examined the effect of endometrioma surgery on ART outcomes as well as ovarian reserve, but there are not enough data on the effect of endometrioma itself and its size as well as laterality on ovarian reserve and fertility outcomes. However, the majority of studies in this field were single-arm without a control group [15, 18], which makes our study one of the firsts in this field to date.

The current work is a retrospective study with all the limitations of other retrospective studies. The sample size herein was small. Additionally, our study lacks sub-classification in patients with unilateral endometrioma and comparison between the affected and healthy ovaries.

A number of papers have evaluated the effect of endometrioma on ovarian reserve due to its inflammatory factors present in the cyst [10, 13]. Some researchers believe that endometrioma, with the increase in intra ovarian pressure, capsule stretching and reduced blood supply, can induce a fall in ovarian reserve and the quality of eggs as a result. Nevertheless, the impact of endometrioma on reproductive outcome is still controversial [12, 20]. (13, 20) In the current study, there was no significant difference in AMH level between the age-matched case and control groups. On the contrary, Radzinsky and Yanushpolsky reported that endometrioma has a negative effect on the number of retrieved oocytes, quality of embryos and the implantation rate in ART cycle [21, 22].

On the other hand, in the study by Ashrafi et al., it was shown that despite the decreasing number of retrieved oocytes in endometrioma compared to that in the healthy control group, live birth rate is similar in both [23].

Regarding the size of the endometrioma and its effect on the ovarian reserve, Schubert et al. showed a decrease in follicle density in the ovarian cortex surrounding the endometrioma compared to other benign ovarian cysts due to the destruction of the ovarian tissue. Menshi et al. also suggested that endometrioma may damage the ovarian tissue even before any operation, which increases with the rise in the size of endometrioma [24, 25]. Despite the existing theories, the effect of endometrioma on reproductive outcome and ART success remains unresolved.

A 2020 study by Alshehre et al. reported a significantly lower number of total oocytes and M2 oocytes retrieved in women with endometrioma compared with the healthy controls [24]. This finding is not consistent with ours, but there is no difference between its results and ours in terms of the quality of embryos, live birth rate (I2 = 67%), clinical pregnancy rate (I2 = 0%), and implantation rate (I2 = 0%) [26].

In accordance with our results, in a systematic review published in 2021 by Dongye et al., the results of 22 studies were reviewed. They concluded that high-quality embryos, embryo formation rate and cleavage rate were similar in women with endometrioma and the healthy control group. Furthermore, in women with the unilateral endometrioma, the quality of the embryos obtained from ovaries containing endometrioma was not significantly different compared to that of healthy ovaries on the opposite side [27]. Hence, endometrioma does not seem to affect the quality of embryos.

In a 2013 study, Benaglia et al. stated that the ovarian hyperstimulation response was significantly lower in women with bilateral endometrioma than that in controls [28]. The number of growing follicles and retrieved oocytes were lower, but no difference was observed in terms of oocyte quality. Clinical pregnancy rate and delivery rate were similar. The final conclusion was that although the presence of bilateral endometrioma at the time of IVF affected the response to hyperstimulation, the quality of retrieved oocytes and the chance of pregnancy did not differ [28, 29]. Most previous works have examined the effect of endometrioma surgery on ART outcome and ovarian reserve rather than the effect of endometrioma itself on the outcome.

In line with our results, in the review by Ashrafi et al. in 2014 indicated that patients with unilateral or bilateral endometrioma of below 3 cm represented similar results as the healthy control group with mild male factor infertility in terms of follicles number, embryo grading (A or B), and pregnancy rate in IVF cycles [23]. However, in 2020, Somigliana et al. concluded that endometriums larger than 4 cm can interfere with ovarian response in IVF cycles [30]. Additionally, Orazov et al. in 2019 reported that egg quality declined in patients with endometrioma of larger than 3 cm. Endometrioma has a negative effect on oocyte quality and ovarian reserve, and has persistent and harmful effects on ovarian reserve after cystectomy [31]. Meanwhile, in agreement with us, in a 2014 systematic review by Barbosa et al., endometriosis in ART-treated patients showed a similar chance of clinical pregnancy and live birth rates compared to that in patients with other infertility-causing issues. No difference was reported in live birth rate among patients with stage 3–4 endometriosis compared to those with stage 1–2 [32].

The fact that an increase in endometrioma size causes a decrease in oocyte quality contradicts the results of our study.

In agreement with our findings, Almog et al. in 2011 concluded that there was no difference in the number of antral follicles and retrieved oocytes between ovaries with endometrioma and healthy ovaries. They also found no correlation between endometrioma size and retrieved oocytes [33]. (The number of antral follicles and oocytes retrieved in patients with endometrioma was equal to that of the control patients, and the presence of ovarian endometrioma in the ovarian stimulation cycle for IVF was not found to be related to the reduction in retrieved oocytes.

Regarding pregnancy outcomes, according to a 2011 study by Bongioanni et al., the ovarian endometrioma presence does not reduce IVF outcome compared to patients with tubal factor infertility, and laparoscopic endometrioma resection does not improve IVF outcomes. However, ovarian response to gonadotropins and antral follicle count may decrease [34]. Similar to our results, in a systematic review conducted by Hamdan et al. in 2015, women with or without endometriosis had similar results in terms of live birth rates, yet there was insufficient evidence to recommend surgery to endometriosis patients before starting an ART cycle [18]. Moreover, in the meta-analysis of Gupta in 2006, he concluded that the clinical pregnancy rate in the endometrioma group is not different compared to that in the control group, and the ovarian response to ovarian hyperstimulation in patients with endometrioma is due to a decrease in the number of follicles compared to that in the control group [35].

In a study conducted by Saeed Alborzi et al., AMH levels significantly decreased after laparoscopic cystectomy for endometrioma, which remained unchanged over time (9 months after the surgery). The patients with bilateral endometrioma had significantly lower AMH levels than their baseline levels after 1 week, 3 weeks and 9 months. Those older than 18 years of age had lower AMH levels after the surgery. The FSH and antral follicle count level increased significantly compared with their baseline levels 3 months following the surgery [17].

A systematic review conducted by Nickkho-Amiry et al. in 2017 examined the effect of surgical management of endometrioma on IVF/ICSI outcomes compared to no treatment. It was concluded that there is no significant difference in pregnancy rate per cycle, clinical pregnancy rate and live birth rate between women with a history of endometrioma surgery and those without that. The outcome of ART cycles in women with endometriosis-related infertility was similar to other women. The final conclusion was that specialists should evaluate the risk of surgical intervention on ovarian reserve before planning a surgery [36].

According to the results of this study, the presence of endometrioma, by itself, does not affect the main result of IVF procedures, including the number and quality of embryos and pregnancy outcome. Accordingly, IVF and embryo preservation and even pregnancy before surgery seems reasonable for patients because after the surgery, there would be a significant and irreversible decrease in AMH level and also response to IVF treatments.

Further clinical and prospective research with high power and sufficient sample sizes are needed to evaluate the effect of endometrioma itself on ovarian function. In addition, further investigation is needed to compare the quality of embryos obtained from affected ovaries and healthy ones in patients with unilateral endometrioma.

Ultimately, it could be concluded that it is better to postpone endometriosis surgery in women who desire pregnancy until there is enough frozen embryos.

## Data Availability

The datasets used and/or analyzed during the current study are available from the corresponding author on reasonable request.

## Abbreviations

IVF: In vitro fertilization
AMH: Anti-müllerian hormone
BMI: Body mass index
FSH: Follicle-stimulating hormone
ART: Assisted Reproductive Technology
ICSI: Intracytoplasmic sperm injection
